# Investigating the relationship between the *SNCA* gene and cognitive abilities in idiopathic Parkinson’s disease using machine learning

**DOI:** 10.1038/s41598-021-84316-4

**Published:** 2021-03-01

**Authors:** Mehrafarin Ramezani, Pauline Mouches, Eunjin Yoon, Deepthi Rajashekar, Jennifer A. Ruskey, Etienne Leveille, Kristina Martens, Mekale Kibreab, Tracy Hammer, Iris Kathol, Nadia Maarouf, Justyna Sarna, Davide Martino, Gerald Pfeffer, Ziv Gan-Or, Nils D. Forkert, Oury Monchi

**Affiliations:** 1 Department of Clinical Neurosciences, Cumming School of Medicine, University of Calgary, Calgary, AB Canada; 2 Hotchkiss Brain Institute (HBI), Cummings School of Medicine, University of Calgary, Calgary, AB Canada; 3 Biomedical Engineering Graduate Program, University of Calgary, Calgary, Canada; 4 Department of Radiology, University of Calgary, Calgary, AB Canada; 5 Montreal Neurological Institute, McGill University, Montreal, QC Canada; 6 Department of Neurology and Neurosurgery, McGill University, Montreal, QC Canada; 7 Department of Medical Genetics, Alberta Children’s Hospital Research Institute, Cumming School of Medicine, University of Calgary, Calgary, AB Canada; 8 Department of Human Genetics, McGill University, Montreal, QC Canada

**Keywords:** Parkinson's disease, Machine learning, Disease genetics, Risk factors

## Abstract

Cognitive impairments are prevalent in Parkinson’s disease (PD), but the underlying mechanisms of their development are unknown. In this study, we aimed to predict global cognition (GC) in PD with machine learning (ML) using structural neuroimaging, genetics and clinical and demographic characteristics. As a post-hoc analysis, we aimed to explore the connection between novel selected features and GC more precisely and to investigate whether this relationship is specific to GC or is driven by specific cognitive domains. 101 idiopathic PD patients had a cognitive assessment, structural MRI and blood draw. ML was performed on 102 input features including demographics, cortical thickness and subcortical measures, and several genetic variants (*APOE*, *MAPT*, *SNCA*, etc.). Using the combination of RRELIEFF and Support Vector Regression, 11 features were found to be predictive of GC including sex, rs894280, Edinburgh Handedness Inventory, UPDRS-III, education, five cortical thickness measures (R-parahippocampal, L-entorhinal, R-rostral anterior cingulate, L-middle temporal, and R-transverse temporal), and R-caudate volume. The rs894280 of *SNCA* gene was selected as the most novel finding of ML. Post-hoc analysis revealed a robust association between rs894280 and GC, attention, and visuospatial abilities. This variant indicates a potential role for the *SNCA* gene in cognitive impairments of idiopathic PD.

## Introduction

Parkinson’s disease (PD) is traditionally associated with motor symptoms. Non-motor deficits have received less attention but are also quite common and can even precede motor symptoms. PD patients can display a wide range of cognitive deficits of various intensity from Mild Cognitive Impairment (MCI: neuropsychological deficits that do not interfere with daily life activities) to dementia^1^. PD patients with cognitive deficits are more likely to develop Parkinson’s disease dementia (PDD) as the disease progresses, compared to patients without such deficits^2^. To address the high prevalence and the inevitable negative impact of cognitive deficits, the Movement Disorders Society (MDS) prepared a task force to assist identification of MCI in PD patients^3,4^. Nevertheless, the underlying mechanisms of PD-MCI development are still obscure and further investigation is required to unravel its nature. At the cellular level, PD is characterized by presence of Lewy Body (LB) deposits in the substantia nigra^5^. LB mainly consists of alpha-synuclein^6^, a protein that is encoded by the *SNCA* gene^7^. *SNCA* is a prominent potential genetic marker in PD due to its involvement in familial PD through point mutations or gene dosage effect^8–10^. *SNCA* is also implicated in a class of disorders called synucleinopathies, which have LB pathology in common, *e.g.* PD (with or without dementia), dementia with LB (DLB), Multiple System Atrophy (MSA), idiopathic REM Behavioral Disorder (RBD). PDD and DLB share numerous similarities contributing to the challenge of distinguishing them from each other^11,12^. The substantial involvement of *SNCA* in PDD and DLB pathology through LB and the similarity of the symptomatology, such as the presence of dementia in these two diseases, indicate a potential role for the *SNCA* gene in cognitive decline of idiopathic PD patients and necessitates further investigation. In recent years, several variants of *SNCA* have been discovered in connection with cognitive impairments or dementia in PD^12,13^. Exploring possible genetic variants associated with cognitive impairments in PD could improve the understanding of the primary biological mechanisms of PD-MCI and identification of patients at risk of cognitive decline. However, given the complexity of PD, further investigation is required to unravel the true involvement of the known and novel genetic variants linked to cognitive deficits in PD using advanced techniques^14^.

In addition to genetic risk factors, structural neuroimaging has been used in PD participants to identify image-based biomarkers of cognitive decline^15–19^. A growing body of evidence supports the use of structural neuroimaging as a biomarker for PD-MCI identification. One example is thinning of frontal and temporal cortices, which has been associated with cognitive decline in PD^15,19,20^. Also, corticometric and volumetric analyses have shown a reduced volume in frontal and limbic regions in PD-MCI compared to PD-nonMCI patients^16,21,22^.

In this study, we aimed to predict global cognition in PD participants using a machine learning approach including genetic, structural neuroimaging, clinical, and demographic data as input. In a second step, using post-hoc analyses, we aimed to identify the relationship between the most novel genetic features and the cognitive profile of PD patients in more depth. Furthermore, we aimed to investigate whether this relationship is specific to global cognition or driven by specific cognitive domains including executive function, attention, visuospatial abilities, memory, and language.

## Results

### Participants

The demographic and clinical characteristics of 101 PD participants included in this study are summarized in Table 1. The mean age of participants was 70 years with a mean disease duration of 6 years. Thirty-two precents of the participants were female (n = 32). Ninety participants were right-handed (89%), six participants were left-handed (6%), and the remaining five participants were identified as ambidextrous (5%). The majority of the participants were of European descent (84%).

**Table 1:** The demographic and clinical characteristics.

Demographic/clinical characteristics	Mean, SD (Min, Max)
Age (years)	70, ± 7.3 (53, 84)
Education (years)	14.4, ± 2.9 (8, 21)
Disease duration (years)	5.7, ± 4.0 (1, 18)
LEDD (mg/d)	869.0, ± 457.6 (200, 2650)
UPDRS-III	19.46, ± 9.41 (4, 53)
EHI	68.9, ± 41.6 (− 100, 100)
Sex (female %)	32%
**Ethnicity**	
European descent	84%
Others	10%
NA	6%
Global cognition Z-score	− 0.27, ± 0.6 (− 2, 1)
Attention Z-score	− 0.24, ± 0.6 (− 1, 1)
Executive function	− 0.43, ± 0.8 (− 3, 1)
Language	− 0.13, ± 0.8 (− 2, 1)
Memory	− 0.15, ± 0.8 (− 2, 1)
Visuo-spatial	− 0.43, ± 0.9 (− 3, 1)

Abbreviations; LEDD = Levodopa Equivalency Daily dosage, UPDRS-III = Unified Parkinson’s disease Rating Scale part III, EHI = Edinburg Handedness Inventory, NA = Not available.

### Machine learning analysis

The global cognition scores were predicted using the 102 features employing the machine learning framework including feature ranking and a support vector regression model. The best model performance predicting global cognition was achieved when including the 11 top-ranked features (Table 2). This resulted in a correlation coefficient of 0.54 and mean absolute error of 0.39. The selected features were (in order of descending importance): sex, rs894280, EHI, UPDRS-III, years of education, five measures of cortical thickness (right parahippocampal cortex, left entorhinal cortex, right rostral anterior cingulate cortex, left middle temporal cortex, and right transverse temporal cortex), and right caudate volume.

**Table 2:** Machine learning analysis results. Features are presented in the right column based on their importance in the machine learning model. The features are presented in a descending order (the most important to the least important).

Cognitive domain	Correlation coefficient, R^2^	Features
Global cognition	0.54, 0.29	Sex rs894280 EHI score UPDRS-III years of Education R parahippocampal-thickness L entorhinal-thickness R rostralanteriorcingulate-thickness L middletemporal-thickness R transeversetemporal-thickness R caudate-volume

Abbreviations: UPDRS-III = Unified Parkinson’s disease Rating Scale part III, EHI = Edinburg Handedness Inventory.

The high-ranked demographic features were further analyzed individually using Pearson correlation or chi-square test. Correlation analysis of sex and EHI score with global cognition were found to be non-significant ($\chi$^2^ = (1, n = 101) 75.59 *p* = 0.46 and R^2^ = 0.005, *p* = 0.50, respectively). Also, UPDRS-III and years of education exhibited significant correlation with global cognition. The UPDRS-III score had a negative correlation while years of education had a positive correlation with global cognition (R^2^ = − 0.26, *p* < 0.001 and R^2^ = 0.07, *p* = 0.007, respectively). The structural measures were tested for correlation with global cognition and two measures showed significant correlation with global cognition: R rostral-anterior cingulate-thickness, and L middle temporal-thickness (R^2^ = 0.08, *p* = 0.004, R^2^ = 0.04, *p* = 0.03, respectively).

### Association of the *SNCA* variant rs894280 and global cognition

Based on the machine learning results, post-hoc analyses were performed to study the association of the alleles of the novel variant rs894280 with global cognition and specific cognitive domains.

Out of 101 participants, 33 had CC genotype, 48 had CT genotype, and 20 had TT genotype. Based on the preliminary analysis of this SNP, participants with the T allele were pooled in one group, resulting in dividing the participants in two allelic groups^23^. The demographic and clinical characteristics were not significantly different between the two allelic groups (Table 3).

**Table 3:** Demographic and clinical characteristics of the two groups of rs894280 variant.

Demographic/clinical characteristics Mean, SD, (Min, Max)	CC n = 33	CT/TT n = 68	Significance
Age (years)	71.5, ± 7.3	69.2, ± 7.3	0.14^a^
Education (years)	14.6, ± 3.2	14.3, ± 2.3	0.66^a^
Disease duration (years)	5.6, ± 4.2	5.7, ± 4.0	0.56^b^
LEDD (mg/d)	767.7, ± 300.9	918.2, ± 511.7	0.31^b^
UPDRS-III	18.14, ± 7.82	20.10, ± 10.09	0.44^b^
EHI	69, ± 40	69, ± 40	0.83^b^
Sex (female%)	33%	31%	0.82^c^
**Ethnicity**			
European descent	88%	82%	0.67^d^
Others	9%	10%	
NA	3%	7%	

Abbreviations; SD = Standard Deviation, LEDD = Levodopa Equivalency Daily dosage, UPDRS-III = Unified Parkinson’s disease Rating Scale part III, EHI = Edinburg Handedness Inventory, NA = Not available.

^a^Student-t test.

^b^Mann–Whitney U test.

^c^Fisher exact test.

^d^Chi-square test.

This variant showed a significant association with global cognition when controlling for UPDRS-III, education, R rostral-anterior cingulate and L middle temporal thickness measures (F (5,95) = 12.17, *p* < 0.001, R^2^ = 0.35). Based on the ANCOVA results, global cognition was significantly different between the CC and CT/TT groups, F (5,95) = 4.20, *p* = 0.04.

According to ANCOVA analysis, each participant’s Z-score of global cognition increased by 0.25 (95% CI = 0.01–0.42) when the participant had CC genotype (i.e. reference sequence) for rs894280. The calculated Hedges’ effect size for rs894280 is 0.4, which represents a medium effect size based on 95% CI.

Association analysis of rs894280 and each cognitive domain was performed using ANCOVA including any demographic/clinical factor with a significant correlation with the domain of interest as covariates. UPDRS-III, education, R rostral-anterior cingulate and L middle temporal thickness measures were included for all domains, except for the visuo-spatial domain for which the EHI score was also added to the model (R^2^ = 0.05, *p* = 0.02). Significance level was set to 0.01 using Bonferroni correction to correct for multiple tests. A significant association was found between rs894280 and the attention domain score (B = − 0.34, *p* = 0.003) with the CC group displaying better attention abilities. Moreover, this variant showed an association with the visuo-spatial domain score (B = − 0.51, *p* = 0.005). Similar to results from global cognition and attention, PD patients with CC genotype had better visuo-spatial abilities compared to the other group (CT/TT). The results of association analysis are summarized in Table 4. A trend was observed for the memory domain (*p* = 0.02) while executive function and language did not show any association with this variant. PD participants homozygous for C allele of rs894280 displayed superior attention and visuo-spatial abilities compared to participants who had one or more T alleles.

**Table 4:** Results of ANCOVA analysis of rs894280, and global cognition and the five cognitive domains.

Cognitive domain Mean, SD, (Min–Max)	CC N = 33	CT/TT N = 68	CI95% _rs894280_	Significance
Global cognition	0, ± 0.5, (− 1, 1)	− 0.4, ± 0.6, (− 2, 1)	0.006–0.42^a^	0.04*
Attention	0, ± 0.5, (− 1, 1)	− 0.4, ± 0.6, (− 1, 1)	0.12–0.56^a^	0.003**
Executive function	− 0.3, ± 0.7, (− 2, 1)	− 0.5 ± 0.9, (− 3, 1)	− 0.10–0.55^a^	0.19
Language	0, ± 0.7, (− 1, 1)	− 0.2, ± 0.8, (− 2, 1)	− 0.16 – 0.44^a^	0.36
Memory	0.2, ± 0.7, (− 1, 1)	− 0.3, ± 0.8, (− 2, 1)	0.07–0.70^a^	0.02
Visuo-spatial	− 0.1, ± 0.6, (− 2, 1)	− 0.6, ± 1, (− 3, 1)	0.16–0.87^b^	0.005**

SD = Standard Deviation, CI 95% = 95% confidence intervals.

^a^UPDRS-III, Education, R rostral-anterior cingulate and L middle temporal thickness measures were entered in the model.

^b^UPDRS-III, Education, R rostral-anterior cingulate and L middle temporal thickness measures and EHI score were entered in the model.

*Significance level was set to 0.05.

**Significance level was set to 0.01, Bonferroni correction.

## Discussion

In this study, we used machine learning to predict global cognition in PD patients and post-hoc analysis to investigate the *SNCA* rs894280 variant as a feature associated with cognitive deficits in PD. Using the RRELIEFF feature selection algorithm and SVR, eleven features were selected as the best predictor of global cognition Z-score in this cohort; sex, rs894280, EHI, UPDRS-III, education, five measures of cortical thickness (right parahippocampal cortex, left entorhinal cortex, right rostral anterior cingulate cortex, left middle temporal cortex, and right transverse temporal cortex), and right caudate volume. The selection of features indicate that these variables are informative for prediction of the global cognition score but the direction for each single feature cannot be easily determined based on the machine learning model. Consistent with the machine learning results, which revealed rs894280 as the only genetic factor informative for PD cognition prediction, further analysis was performed on the association of this variant and global cognition. The results suggest that this variant is associated with differences in global cognition, as well as attention and visuo-spatial domains in our cohort, with a medium effect size (Hedges’ g = 0.4).

The RRELIEFF approach was used to remove redundant and non-informative features and to select the optimal subset of features. This feature selection method has been used for optimal selection of genetic features in previous studies^24–27^. The SVR was implemented to build the model based on the selected features in order to evade the collinearity issue of the features. The SVR model has been used previously to model PD diagnosis and progression^28,29^ but the current combination used in this study has not been applied before specifically in investigation of cognitive deficits in PD with similar set of inputs.

Except for rs894280, all other features used in the optimal regression model have been reported in different studies to be associated with cognitive decline in PD patients. There is a substantial body of evidence on the role of sex in cognitive decline in PD, with male patients showing greater risk of cognitive impairments^30–32^. However, it also needs to be mentioned that several other studies reported no evidence of the impact of sex in cognitive decline in PD^4,33^. The reason for these conflicting results remains speculative but could be related to the sample size. EHI is a well-known screening tool to determine handedness^34^. The correlation between the dominant hand and the side of motor symptoms onset has been suggested by several studies^35–37^. This relationship might extend to the cognitive impairments in PD as some studies suggest^38,39^.

The severity of motor symptoms has been suggested to be one of the strongest risk factors for cognitive deficits in idiopathic PD^4,40–42^. UPDRS-III is one of the most widely used screening tools for the severity of motor symptoms in PD and can accurately and efficiently note the presence and progression of those symptoms^43^. In line with the findings of this study, other studies have also found evidence for a connection between the severity of motor symptoms and emergence of cognitive decline^44,45^. The number of years of education was also found to be predictive of global cognition in our cohort. Similarly, a large body of evidence reported a negative correlation between higher education level and the likelihood of cognitive impairments in PD patients^4,30,33^. It has been suggested that education has a role in preserving the cognitive reserve in PD patients at risk of cognitive decline^46^.

The structural biomarkers of cognitive decline in PD have been investigated extensively using different techniques^33,47,48^. The right parahippocampal gyrus, the top anatomical feature identified in this study, has been reported as one of the main brain regions showing significant Dopamine receptor (D2) binding reduction in PD patients^49^. Another machine learning study reported the parahippocampal region as a top feature showing the highest correlation with the motor score in PD^50^. Similarly, this region was also identified as one of the top features in Alzheimer’s disease (AD). These findings suggest a more general function of the parahippocampal region in neurodegenerative diseases given its prominent role in memory^51^. The entorhinal cortex was previously reported as one of the main brain regions allowing a fine distinction between PD-MCI and PD-nonMCI patients^52^. The right entorhinal volume was observed to be positively correlated with memory abilities in early drug-naïve PD-MCI patients^52^. Additionally, cortical thinning of the entorhinal region was found to correlate significantly with memory impairments in PD patients^53^. The anterior cingulate cortex is another ROI associated with cognitive impairment in PD. A large body of evidence indicates a link between PD-MCI cognitive status and the anterior cingulate^49,54–56^. These findings are in accordance with the results of the present study and indicate a potential relationship between this region and cognition in PD. The last anatomical feature predictive of global cognition was the right caudate volume. The caudate nucleus is one of the chief regions in PD pathology and extensive loss of neurons in this nucleus was reported in association with cognitive impairment and dementia in PD^57–60^.

We used a combination of known genetic risk factors (H1 *MAPT*, $\varepsilon$4 *APOE, COMT p.Val158Met, DAT1 VNTR, BDNF p.Val66Met*) and novel genetic variants to predict the global cognition in this cohort^12,30,60^. The rs894280 was selected as the novel finding for the post-hoc analysis because of its importance in the machine learning model. Ranked as the second top feature, this variant could present a meaningful role in prediction of global cognition in this cohort. This finding was in contrast with the known genetic risk factors included in this study which were not selected by the machine learning model. This variant is an intronic polymorphism located on the 5′ region of *SNCA* gene and was initially reported in association with dementia with Lewy bodies (DLB)^61^. The role of *SNCA* gene mutations in familial PD has been known for decades. However, new data suggest a role for this gene in cognitive deficits and dementia in idiopathic PD^12,13,62,63^. A recent study indicated association between several *SNCA* variants and worse performance in Mini Mental state examination (MMSE) in PD patients^63^. Similar association was reported on the association of several *SNCA* variants and PDD^12^. A microsatellite (Rep1) is located on the 3′ region of the *SNCA* gene and has two common alleles (short repeat and long repeat). The long repeat allele of Rep1 seems to increase *SNCA* transcription and was reported to be linked to lower MMSE scores in PD patients^62^. On the other hand, the 5′ region of the *SNCA* gene was considered as a haplotype specific for DLB and not PD. This evidence was further supported by another study investigating the *SNCA* role in both DLB and PD^12^. Both PDD and DLB are classified as synucleinopathies and share substantial similarities in symptoms and pathology, to the point that the exact differentiation of these two disorders clinically and pathologically are still a matter of debate^64^. The rs894280 has been reported in association with both DLB and PDD and this might suggest a more general role for this variant in LB pathology.

Furthermore, rs894280 is in linkage disequilibrium (LD) with rs1348224 with comparable odds ratio (D’ = 1.0, R^2^ = 1.0). The rs1348224 variant was previously reported in association with PDD, surviving multiple testing in a sample of 1492 PD patients^12^. Moreover, a strong correlation was reported between rs894280 and the Hopkins Verbal Learning Test-Revised (HVLT-R) total recall in PD patients (*p* = 6.1 × 10^–4^), and it displayed the strongest relationship with cognitive abilities out of 39 *SNCA* variants included in that study. However, this association did not survive after correction for multiple comparisons. This could indicate a role for rs894280 in PD cognitive abilities, especially in the memory domain^65^. A Brazilian study found cognitive impairments in PD patients carrying T allele of rs2583988 of *SNCA*. The rs2583988 is in a strong LD with rs894280 (D′ = 1.0, R^2^ = 0.40 in European descendent populations), which further indicates a possible role for rs894280 in cognitive decline of PD patients^13^.

Deficits in the attention, visuo-spatial, and memory domains are frequently reported in PD-MCI patients^3,66,67^. Association of rs894280 with impairments in these domains in idiopathic PD patients may indicate a role for this variant in the development of such deficits. Specifically, this SNP might be connected to visuo-spatial abilities given that attention measures used in this study have a prominent visuo-spatial component. Studies have shown that attention measures with a visual component can tap on to visuo-spatial abilities^68^. Out of three attention measures used in this study, two of them; Trail A and Symbol Span have the required component to engage both visuo-spatial and attention suggesting a potential role for this SNP in connection to visuo-spatial abilities.

We did not observe any association between executive function and language domains and rs894280 in our cohort. A possible explanation for this could be that executive function impairments involve the frontal-striatal areas while most of the cognitive deficits identified in this study are focused in more medial temporal lobe and posterior cortical regions^30^. Although deficits in the language domain are reported in association with dementia in PD^30^, this SNP did not show any link to language abilities in this cohort.

This study had some limitations that should be mentioned. We used a machine learning approach in this study in an effort to capture the underlying complex patterns of the various input features and focused on the most unique and relevant features for further investigation. These findings are preliminary and need replication in larger cohorts. The present cohort size was small for a genetic analysis, but our results displayed a fair level of robustness, in a cohort that is extensively phenotyped and well-characterized. These results need to be replicated in a larger cohort with higher number of genetic variants to avoid missing effect of other potential risk variants before a definite conclusion can be inferred on this specific variant and cognitive impairment in PD.

In conclusion, using machine learning, we found that rs894280 in *SNCA* was one of the top features predictive of cognition in PD patients. Further analysis in the same cohort revealed association of this variant (CC genotype) with attention and visuo-spatial abilities in PD patients with a trend in the same direction for memory abilities. These results indicate a potential involvement of *SNCA* variant rs894280 in the cognitive deficits and even dementia in idiopathic PD patients.

## Methods

### Participants

101 PD patients at Hoehn and Yahr stages II-III were recruited. All patients had a confirmed diagnosis of idiopathic PD by a movement disorder clinic neurologist, meeting the UK brain bank criteria for idiopathic PD. All participants were responsive to dopaminergic medications and took their usual dosage of medications during all study visits. None of the participants were asked to modify their medications for this study. Exclusion criteria were: 1) any neurological disorder other than PD, 2) alcohol dependency, 3) history or presence of a severe psychiatric disorder, and 4) cerebrovascular disorders. The severity of motor symptoms was assessed by a trained professional using the motor section of the Unified Parkinson’s Disease Rating Scale (UPDRS-III). Levodopa Equivalency Daily Dosage (LEDD) and disease duration of all participants were calculated by a research nurse.

All participants provided written informed consent according to the declaration of Helsinki and the study was approved by the Conjoint Health Research Ethics Board (REB14-2463) at the University of Calgary, AB, Canada. All methods were carried out in accordance with the relevant guidelines and regulations.

### Genotyping

A blood sample was collected from each participant and DNA was extracted using an isopropanol-based protocol. DNA samples were screened for several Single Nucleotide Polymorphisms (SNP) using TaqMan genotyping assays on a C-1000 Touch Thermal Cycler. The list of all SNPs and TaqMan assays investigated in this study are shown in Table 5. TaqMan assay reading was done using Applied Biosystem Quantstudio Flex 7 Real-Time PCR system (Fisher Scientific) according to the manufacturer’s instructions. TaqMan (assays) results were analyzed using Bio-Rad CFX Maestro software. The 40 bp Variable Tandem Repeats (VNTR) located on the 3′ region of Solute Carrier 6 family 3 *(SLC6A3)* was amplified using PCR (30 s at 95 °C , 36 cycles of (95 °C for 15 s, 60 °C for 30 s, 70 °C for 60 s), 68 °C for 5 min and 4 °C for hold ) on a C-1000 Touch Thermal Cycler (Biorad), using the primers and protocol described previously^69^. PCR products were mixed with loading dye and loaded on 2% agarose gel containing gelstar and run at 120 V for 30 min followed by 60 min at 100 V. A 100 bp DNA ladder (Biohelix, DM 001-R500F, FroggaBio) was loaded on each gel to determine the molecular size of PCR products. The length of PCR products was captured using the Chemidoc Imaging System (Biorad).

**Table 5:** The list of the TaqMan genotyping assays and the primers used for genotyping.

Gene	Variant	TaqMan genotyping assay
*SNCA*	rs7689942	C-28994912-10
	rs894280	C-8933128-10
*APOE*	rs7412	C-904973-10
	rs429358	C-3084793-20
*COMT*	rs4680	C-25746809-50
*MAPT*	rs393152	C-2265265-10
*BDNF*	rs6265	C-11592758_10
SLC6A3*	VNTR 9/10 R	F-TGTGGTGTAGGGAACGGCCTGAG R-CTTCCTGGAGGTCACGGCTCAAGG

*SLC6A3 VNTR was genotyped using custom made primers. For details please refer to methods.

### Neuropsychological assessment

All participants completed a comprehensive cognitive assessment. The cognitive battery applied in this study consists of tests and measures covering five cognitive domains: executive function, memory, attention, visuo-spatial, and language. The full list of cognitive tests is shown in Table 6. All tests were scored by a trained psychometrist. The cognitive tests were first scored using the test makers manual, which details specific parameters to evaluate an examinee's performance. The total raw score is then converted to a standardized score by comparing the examinee's score to other healthy controls matched for age. All neuropsychological tests that were corrected for age, and most were corrected for years of education, and sex.

**Table 6:** The neuropsychiatric battery.

Cognitive domain	Cognitive test/measure
Attention	Trail A Weschler Memory Scale (WMS) symbol span Digit-span Forward
Executive function	Hayling 2 Brixton Trail B Stroop Colour/Word Clock drawing command
Language	Animals Actions Boston Naming test (BNT)
Memory	Rey Complex Figure Test (RCFT) delay recall Hopkins Verbal Learning Test (HVLT) Retention HVLT Recognition Logical memory delayed recall
Visuo-spatial	RCFT copy Hooper Visual Organizational Test

The measures corresponding to the same cognitive domain were averaged to obtain the average Z-score for each of the cognitive domains. The global cognition Z-score was calculated by averaging all cognitive domains’ Z-scores. The Edinburgh Handedness Inventory (EHI) was administered to the participants and scored to identify each participant’s dominant hand.

### MRI acquisition

Each participant had an MRI scan within two weeks of the neuropsychological assessment using the GE Discovery 750 3 T MRI at the Seaman Family Imaging Centre at the University of Calgary, Calgary, Alberta, Canada. A high-resolution T1-weighted 3D inversion recovery prepared fast spoiled gradient recalled (IR-FSPGR) sequence was acquired for each participant (repetition time = 7.176 ms, echo time = 2.252 ms, flip angle = 10°, acquisition matrix = 256 × 256, voxel size = 1 × 1 × 1 mm^3^, 172 slices).

### Cortical thickness and subcortical volume

Freesurfer (http://surfer.nmr.mgh.harvard.edu/; version 6.0.0) was used to perform cortical thickness and subcortical volumetric analyses. The analysis was performed following the procedure detailed in prior publications^70–72^. Cortical segmentation was performed automatically and upon visual inspection, appropriate manual adjustments were made. The manual editing was carefully performed in accordance with the Freesurfer manual in several steps (https://surfer.nmr.mgh.harvard.edu/fswiki/Edits). The errors resulting from imperfect intensity normalization were corrected by inserting control points and where appropriate, extraneous tissue were removed from the brain volumes (wm.mgz and brainmask.mgz). A total of 29 segmentation results were manually edited. Cortical thickness was computed for 68 Regions-of-Interest (ROIs) using the Deskian-Killiany brain atlas in Freesurfer^73^. Furthermore, the mean cortical thickness was calculated for each hemisphere.

Subcortical volumetric measures were computed from eight regions per hemisphere including caudate nucleus, putamen, pallidum, nucleus accumbens, hippocampus, amygdala, thalamus, and ventral diencephalon, plus the brain stem. All subcortical volumetric measures were corrected for the intracranial volume.

### Machine learning

The input features used for machine learning were sex, age, EHI score, years of education, years of disease duration, LEDD, UPDRS-III, rs6265, rs7689942, rs894280, rs7412, rs393152, rs429358, rs4680, and *SLC6A3* VNTR. Furthermore, the following imaging measures were included: cortical thickness measures (68 ROIs), subcortical volumetric measures adjusted for the intracranial volume (17 ROIs), and 2 measures of global thickness. In total, 102 measures were available per participant for the machine learning analysis to predict the outcome variable, the global cognition Z-score.

The machine learning analysis consisted of two main steps: (1) feature ranking and selection, and (2) regression analysis. The feature ranking was employed to rank the 102 features (8 genetic, 87 neuroimaging, 3 clinical, and 4 demographic) based on their contribution to the outcome variable and to select the most efficient combination of features that can predict the outcome variable of the regression problem. Reducing the number of features is essential for improving model performance by eliminating features that are redundant and non-informative. In this study, the RRELIEFF feature selection algorithm was used for this purpose^74^. In the next step, the machine learning regression modelling was performed based on the ranked features using a Support Vector Regression (SVR) model with a polynomial kernel. The SVR is, in principle, very similar to the support vector machine classification models with slight differences for the adaption to a regression style problem^75^. More precisely, an SVR model is built based on only a subset of training data within the predefined margins that minimize the generalization error. Therefore, the data is first transformed into a higher dimensional space employing the polynomial kernel, thereby allowing linear models to fit the training data. The SVR model was used in this study for regression modeling as it is less likely to overfit the data compared to other models, i.e. SVR is a model with adequate generalization capabilities and good prediction accuracy.

The least informative feature was iteratively removed from the set of ranked features until only two features were left for model training to identify the optimal subset of features. The model performance was evaluated for each iteration using the root mean squared error comparing the predicted with true observations. Finally, the model with the optimal feature subset was further evaluated using additional metrics including the coefficient of determination (R^2^) and the correlation value. The coefficient of determination quantifies the amount of variance in the outcome variable that is explained by the selected features in the model. A nested leave-one-out method of the cross-validation was employed through the feature selection and regression in which the number of model validation was set to N where N is equal to the number of participants in the sample. At each validation test, one participant is used to test the model while N-1 participants were used to train the model. All metrics reported for machine learning were attained by averaging the metrics of these N models. This method was used to overcome the small sample size and to prevent double-dipping.

### Statistical analysis

Statistical analyses of continuous variables were performed using either a student-t test or Mann–Whitney U test based on the data normality. The Fisher exact test was used for categorical variables.

The post-hoc statistical analysis was designed in compliance with the machine learning results. Pearson correlation test was used to select independent factors correlated with the target feature. ANCOVA was used to explore the allelic group differences in the rs894280 variant of the *SCNA* gene with regards to global cognition. Demographic and clinical factors that were significantly correlated with a cognitive domain score of (attention, language, etc.) were included as covariates in the ANCOVA to control for them. A value of *p* < 0.05 was considered significant for the single tests, and Bonferroni correction was used to correct for multiple testing. The chi-square test was used to explore association of rs894280 with other genetic variants available in this cohort. All statistical tests were performed using IBM Corp. Released 2019. IBM SPSS Statistics for Mcintosh, Version 26.0. Armonk, NY: IBM Corp.
